# Examination of Correlates to Health-Related Quality of Life in Individuals with Fragile X Syndrome

**DOI:** 10.3390/brainsci10040213

**Published:** 2020-04-04

**Authors:** Marika C. Coffman, Rebecca C. Shaffer, Lauren M. Schmitt, Kelli C. Dominick, Ernest Pedapati, Angel Wang, Elizabeth Berry-Kravis, Nicole Tartaglia, Craig A. Erickson

**Affiliations:** 1 Division of Developmental and Behavioral Pediatrics, Cincinnati Children’s Hospital Medical Center, Cincinnati, OH 45229, USA; marika.coffman@cchmc.org (M.C.C.); lauren.schmitt@cchmc.org (L.M.S.); 2 Department of Pediatrics, University of Cincinnati College of Medicine, Cincinnati, OH 45267, USA; 3 Division of Child and Adolescent Psychiatry, Cincinnati Children’s Hospital Medical Center, Cincinnati, OH 45229, USA; Kelli.Dominick@cchmc.org (K.C.D.); Ernest.Pedapati@cchmc.org (E.P.); craig.erickson@cchmc.org (C.A.E.); 4 Department of Psychiatry and Behavioral Neuroscience, University of Cincinnati College of Medicine, Cincinnati, OH 45267, USA; 5 Division of Child Neurology, Cincinnati Children’s Hospital Medical Center, Cincinnati, OH 45229, USA; 6 Departments of Pediatrics, Rush University Medical Center, Chicago, IL 60612, USA; Angel_wang@rush.edu; 7 Departments of Pediatrics, Neurological Sciences, Biochemistry, Rush University Medical Center, Chicago, IL 60612, USA; Elizabeth_Berry-Kravis@rush.edu; 8 Developmental Pediatrics, Children’s Hospital Colorado, Aurora, CO 80045, USA; nicole.tartaglia@childrenscolorado.org; 9 Division of Developmental Pediatrics, Department of Pediatrics, University of Colorado School of Medicine, Aurora, CO 80045, USA

**Keywords:** fragile X syndrome, health-related quality of life, quality of life, adaptive behavior

## Abstract

Health-related quality of life (HRQoL) is a multidimensional concept involving physical, psychological, social, and cognitive aspects of life. Individuals with Fragile X syndrome (FXS) experience a life-long disorder that impacts the HRQoL of the affected individual and their family. Thus, HRQoL may be an important outcome measure following intervention. However, it is yet not known whether HRQoL concerns relate to observed impairments in FXS. In the present study, we examined the nature and degree of association between HRQoL and established measures of functioning in FXS using the Parent Report for Children version of the PedsQL 4.0 Generic Core Scales and Cognitive Functioning Scale. We observed significant relationships between HRQoL a nd measures of adaptive behavior, maladaptive behaviors, and social functioning. The present study has implications for treatment outcomes for clinical trials in FXS.

## 1. Introduction

Fragile X syndrome (FXS) is a complex neurodevelopmental disorder, associated with a heterogeneous presentation of significant impairments across cognitive, language, sensory, behavioral, and emotional domains. Symptoms of autism, or a comorbid diagnosis of autism spectrum disorder (ASD), are also common in FXS. Given the complex phenotype, individuals with FXS often present to clinical settings for numerous concerns, including intellectual disability (ID), hyperarousal to sensory stimuli, anxiety, aggression, sleep issues, hyperactivity, and social and communication difficulties, among others [1]. These common clinical issues associated with FXS can have a lifelong impact on the quality of life (QoL) of the child and family, indicating QoL may be an important outcome measure following intervention [2]. However, it is not known whether the level of QoL concerns is associated with severity of impairments in FXS.

The United States Food and Drug Administration (FDA) has stated that QoL is a general concept, implying that evaluation of the effect of all aspects of life on general well-being is too general to be considered appropriate when evaluating the efficacy or effectiveness of treatment. Therefore, there is a need for development and examination of more specific concepts of QoL in FXS if this area is to be addressed in future treatment studies. Health-related quality of life (HRQoL) is a more specific, multidimensional concept involving physical, psychological, social, and cognitive aspects of life [3,4]. In children, examining HRQoL and global functioning is particularly important for the evaluation and optimization of their health and development [5,6,7]. This has led to the FDA affirming that HRQoL, as defined above, can be evaluated as an outcome measure of change with treatment.

In psychiatric conditions, HRQoL is associated with child psychopathology across disorders [8] and has been utilized as an outcome measure for major depressive disorder [9] and panic disorder [10]. For example, better HRQoL after treatment for depression was associated with reduced remission rates and fewer somatic symptoms [9]. Similarly, improvements in HRQoL are associated with reduced anxiety-related avoidance, as well as remission of anxiety-related disorders [10]. 

The present study builds upon prior work examining HRQoL in youth with FXS through an online survey [2]. In this prior work, the highest HRQoL was observed in physical functioning, followed by emotional, school, and social functioning. Moreover, higher HRQoL was reported in females compared to males. The current study extends this work in several meaningful ways, including with (1) thorough characterization of participants through an in-clinic visit, and (2) additional characterization with broader behavioral information. First, we examined whether parents who bring their children in for testing differed in their ratings of their child’s HRQoL compared to those who completed the measures online. Second, we examined whether HRQoL in FXS is associated with (a) adaptive behavior, (b) social behavior, and (c) maladaptive behaviors. Finally, we examined sex differences in HRQoL, as sex differences are a hallmark of FXS, with males with FXS demonstrating greater impairment [11].

## 2. Methods

### 2.1. Participants

One hundred and fifty-five parents completed questionnaires including the PedsQL on their child as part of an extension of the Fragile X Online Registry With Accessible Research Database (FORWARD) using a multisite design to collect additional essential longitudinal phenotyping data in individuals with FXS through a comprehensive core battery of outcome measures administered yearly. Families participated from one of three sites, the Cincinnati Children’s Hospital Medical Center, Rush University, and the Children’s Hospital of Colorado. IRB approval was obtained for the study at all sites (Cincinnati Children’s Hospital Medical Center Institutional Review Board (IRB #: 2012-2445); Colorado Multiple Institutional Review Board (IRB#: 15-1538); Rush University Office of Research Affairs (IRB#: 08121202)) and informed consent was obtained from parents. Parents completed the PedsQL as proxy reporters for their children. In addition to completion of the study questionnaires, parents reported the age in years and the sex of their child. Participants’ ages ranged from 1.6–20.94 years old (mean age = 15.37; SD = 11.11). Consistent with the ratio of males to females with full mutation FXS, more surveys were completed by parents of males with FXS (*n* = 112, 72.25%) compared to females with FXS (*n* = 43, 27.75%). The Stanford–Binet Intelligence Scales, fifth edition (SB-5) were fully administered to 113 of the participants with complete PedsQL data. Demographic data are shown in Table 1. 

### 2.2. Measures

#### 2.2.1. PedsQL

The parent report for children versions of the PedsQL 4.0 Generic Core Scales and Cognitive Functioning Scale (PedsQL) include twenty-nine items measuring five core dimensions of health: physical functioning (8 items; example: “problems with… walking more than one block”), emotional functioning (5 items; example: “problems with… feeling afraid or scared”), social functioning (5 items; example: “problems with…getting along with other children”), school functioning (5 items; example: “problems with…paying attention in class”), and cognitive functioning (6 items; example: “problems with…difficulty remembering what people tell him/her”). The psychosocial functioning subscale is comprised of the emotional, social, and school functioning items. The parent report versions used in this study assess parents’ perceptions of their child’s HRQoL by asking how much of a problem their child has had with specific activities within each dimension of health in the past month (for Rush University and the Cincinnati Children’s Hospital Medical Center) and past week (for the Children’s Hospital of Colorado, used the acute version of the PedsQL). An ANOVA was conducted for site by subscale to test for differences by site. No significant differences by site were observed; therefore, data from each site was included in all analyses. 

All items across the three measures used are scored, using a five-point Likert scale: 0 = never a problem, 1 = almost never a problem, 2 = sometimes a problem, 3 = often a problem, 4 = almost always a problem. Based on standard scoring practice, all responses were reverse-scored and then linearly transformed to a 0–100 scale (0 = 100, 1 = 75, 2 = 50, 3 = 25, 4 = 0). Subscale scores were determined as the sum of the item scores divided by the number of items answered (to account for missing data). Higher scores indicated higher QoL/functioning. Missing values for some subscales resulted in completed data for the following subscales: total (*n* = 153), physical (*n* = 155), social (*n* = 150), school (*n* = 136), and emotion (*n* = 153).

#### 2.2.2. Vineland Scales of Adaptive Behavior

The Vineland-3 [12] parent interview evaluates adaptive behavior functioning and is widely utilized across a range of developmental disabilities. The scales are organized into three domains including communication, daily living skills, and socialization. The scale is also used to ask for the child’s abilities and skills, as well as their adaptive skills. The Vineland-3 domain-level parent/caregiver form consists of 180 questions, where the parent or caregiver can circle 2 for usually or often, 1 for sometimes or 0 for never. Missing values for some subscales resulted in completed data for the following subscales: communication (*n* = 127), daily living skills (*n* = 126), and socialization (*n* = 127).

#### 2.2.3. Social Responsiveness Scale

The SRS-2 [13] is aimed at assessment of deficits in social behavior related to autism spectrum disorder (ASD). The SRS-2 consists of 65 questions scaled from 1 (not true) to 4 (almost always true). The social awareness subscale measures an individual’s ability to recognize social cues of others by the use of eight questions. The social cognition subscale uses 12 items that address interpretation of social behavior. The social communication subscale has 22 items and assesses reciprocal communication in social situations. The social motivation subscale has 11 items that assess the degree to which an individual is motivated to participate in social interactions with others. The restricted interests and repetitive behavior subscale measures stereotypy and circumscribed interests using 12 items. Data were available for 128 participants for this questionnaire.

#### 2.2.4. Aberrant Behavior Checklist

The ABC-C is a 58-item caregiver report questionnaire on behavior difficulties commonly seen in individuals with developmental disabilities (DD) [14]. There are five subscales derived by factor analysis: irritability, social withdrawal/lethargy, stereotypy, hyperactivity, and inappropriate speech. The ABC has been extensively used in psychopharmacological studies of ASD and other developmental disorders [15]. Caregivers rate the severity of behaviors (i.e., temper tantrums/outbursts) on a 4-point scale ranging from 0 = not a problem to 3 = the problem is severe in degree. The ABC has been used previously as an indicator of irritability and behavioral impairment. The ABC was completed for 83 children in the present study. In addition, a total score can be calculated. Factor analysis of the ABC supported a six-factor structure for use in FXS [16]. Therefore, the six-factor model was used in the present study which includes an additional sixth factor, social avoidance.

### 2.3. Statistical Analyses

First, to examine whether the values observed in the present study were comparable to a previously reported study on the PedsQL from survey data, independent samples *t*-tests between the prior study [2] were performed, as well as Pearson’s correlations between subscales. Pearson’s correlations by sex were conducted to examine potential sex differences. Finally, to examine the relationship between HRQoL and psychosocial behaviors, Pearson’s correlations were conducted between subscales of the PedsQoL and the following psychosocial scales: Vineland Adaptive Behavior Scales, the Social Responsiveness Scale, and the Aberrant Behavior Checklist.

All analyses were conducted in R Studio [17].

## 3. Results

Independent samples *t*-tests between the current sample and previously reported scores revealed that parents who reported on their child’s quality of life through an online survey reported significantly lower QoL than parents who brought their children with FXS in for a clinic visit (Table 2). In particular, there were significant differences between scores on the social scale (*t*(516) = 2.53, *p* = 0.01), school scale (*t*(516) = 2.15, *p* = 0.03), and emotional scale (*t*(516) = 2.35, *p* = 0.02). Parents did not report significant differences in functioning on the physical scale (*t*(516) = 1.3, *p* = 0.19). Despite significant differences between means, a similar pattern of HRQoL emerged across studies. The greatest HRQoL was reported in the physical and emotional scales, followed by school functioning, with social HRQoL rated the lowest consistently.

### 3.1. Correlations between Subscales on HRQoL

Significant correlations were observed between all subscales on the PedsQL. The physical scale was correlated with the psychosocial scale (*r*(151) = 0.63, *p* < 0.001), the emotional scale (*r*(151) = 0.42, *p* < 0.001), the school scale *(r*(134) = 0.51, *p* < 0.001), the social scale *(r*(148) = 0.56, *p* < 0.001), and the total score (*r*(151) = 0.87, *p* < 0.001). The emotional scale was correlated with the social scale *(r*(147) = 0.43, *p* < 0.001), the school scale *(r*(132) = 0.37, *p* < 0.001), and the psychosocial scale *(r*(149) = 0.73, *p* < 0.001). The social scale was correlated with the school scale *(r*(131) = 0.57, *p* < 0.001) and the psychosocial scale *(r*(148) = 0.89, *p* < 0.001). Both males and females demonstrated similar patterns of correlations between subscales on the PedsQL.

Because prior work found an association between age of the participant and QoL scores [2], correlations between age and subscales on the PedsQL were also examined. The only significant correlation was observed between age and the school scale *(r*(134) = 0.21, *p* = 0.01)*,* such that as age increased, school QoL also increased. No other significant correlations were observed between age and PedsQL (*p*s > 0.05).

### 3.2. Associations between Child QoL and Adaptive Functioning

Correlations between the PedsQL scores and the Vineland Adaptive Behavior Scales (Vineland 3) in Table 3 show significant associations. Overall, the strongest correlations for the PedsQL were in the physical scale, which was significantly associated with the socialization (*r*(125) = 0.433, *p* < 0.001), communication (*r*(125) = 3.64, *p* < 0.001), and daily living (*r*(125)=4.68, *p* < 0.001) scales on the Vineland-3. Total scores on the PedsQL were also highly correlated with standard scores on the Vineland-3, with the greatest association with socialization (*r*(123)=0.36, *p* = 0.001), followed by daily living skills (*r*(123) = 0.31, *p* 0.001), and communication skills (*r*(123) = 0.24, *p* = 0.006). The social scale of the PedsQL showed significant associations with Vineland 3 between the social scale (*r*(121) = 0.28, *p* = 0.001), and the daily living skills (*r*(121) = 0.21, *p* = 0.018). The school scale of the PedsQL demonstrated a significant correlation with the socialization scale of the Vineland (*r*(106) = 0.28, *p* = 0.006).

### 3.3. Associations between Child QoL and Socially Aberrant Behaviors

Correlations between all scales on the PedsQL scores and the Social Responsiveness Scale show significant associations, such that better QoL was associated with less social impairment. Specifically, the higher total scores on the PedsQL were associated with less social impairment on the SRS (*r*(126) = −0.656, *p* < 0.001).

Next, the relationship between child’s quality of life and aberrant behavior domains was examined. Across all subscales, greater parent-reported quality of life was associated with reduced aberrant behaviors (all *p’s* < 0.05). Correlations between subscales are reported in Table 4.

## 4. Discussion

This study describes HRQoL and its associations among the dimensions of health in youth with FXS, as well as the associations between HRQoL and standard measures of functioning. Notably, parents reported less impaired HRQoL in their child with FXS in the present sample compared to those who completed the measure online. However, we found a similar pattern of functioning between subscales as in prior research [2]. We also found that parent report on the PedsQL was significantly correlated with adaptive functioning, social impairment, and aberrant behavior.

Here, we found that parents of children with FXS who brought their children in for a characterization study associated with a Fragile X clinic reported overall higher HRQoL than parents who completed the same questionnaire online. Children who are able to attend clinic visits may have higher functioning generally than those who complete an online survey. Alternatively, differences between samples may be attributable to perceived parental competence. For example, parents who brought their child in for a study visit may feel more comfortable bringing their child in for an elective visit. Despite differences in parent-reported HRQoL, the present study found a similar pattern of HRQoL, with the highest HRQoL in physical and emotional functioning, followed by school functioning, then social functioning. These results indicate a consistent pattern of HRQoL concerns for parents of children with FXS.

Children in the present study were also significantly older than children in the online study. This finding adds further data to support prior findings that increased age is associated with better HRQoL [2]. This is also consistent with research on other developmental disabilities, including ASD, showing that parents have reduced stress as their child ages, indicating accommodation by parents to their child’s symptoms [18,19,20,21]. One possibility for this finding is that consistent involvement with treatment (e.g., pharmacological, educational, speech, behavioral) may itself improve HRQoL. It is also possible that with age, children with FXS mature and are more functional. However, there are currently no longitudinal studies on HRQoL in individuals with developmental disabilities, indicating a need to examine the relationship between age and HRQoL further.

QoL measures for clinical trials are thus far unproven. Instead, previous trials in FXS have utilized proxy reports of QoL, such as of adaptive functioning and maladaptive behaviors, including the Vineland 3 and ABC, and the Parenting Stress Index [8]. The present study identified a number of significant relationships between parental report on the PedsQL and other validated measures utilized in clinical trials in FXS. For adaptive behavior, we observed associations between total HRQoL functioning and each domain on the Vineland 3, including socialization, communication, and daily living skills. Parents who reported better adaptive functioning also reported better HRQoL overall. The strongest relationship was observed consistently in the physical domain on the PedsQL. Better physical QoL in FXS likely manifests as greater self-care ability, which was reflected in adaptive behavior. Significant relationships were also observed between social adaptive behaviors (via the Vineland 3), and the social domain on the PedsQL. Better socialization skills as measured by the Vineland 3 likely leads to enactment of these skills across settings, as measured by the PedsQL.

Moreover, parents reported greater social impairment and more problem behaviors in children who had poorer HRQoL on the SRS and ABC, respectively. This was observed across all domains of both the PedsQL and the SRS and ABC. The PedsQL is significantly associated with parent and family factors, including parenting stress [8], and significant associations between parental QoL and family QoL have been observed in FXS [2]). Irritability and aggression can result in injuries and stress for caregivers and require a more restrictive educational and/or living environment [22]. Therefore, it is likely that greater irritability in children with FXS has a significant, negative impact on both the child’s QoL and also the family system, but this will need to be explored in future studies.

Notably, we did not observe significant sex differences in HRQoL domains. Although males and females with FXS are known to differ with regard to co-occurring conditions [11], it appears that HRQoL is evenly impacted across genders.

The present study addresses several weaknesses from the previous study. First, the present involved significantly more phenotyping, including confirming the diagnosis of FXS in all participants. We also included an additional parental report on child functioning, allowing for a more complete picture of HRQoL. However, the present study also contains limitations that should be noted. We did not include a control group. It will be important for future studies to include a matched control group, including alternative neurodevelopmental disorders. We were unable to have participants complete the PedsQL about themselves, as the majority of individuals with FXS lack the ability to self-report via a questionnaire. Finally, further work with significant additional data collection and substantial additional analyses will be required to thoroughly establish the psychometric properties of the PedsQL in FXS, including work to establish test–retest reliability, validity, and factor structure in this population.

FXS is a lifelong disorder that impacts children and families across contexts. Examining QoL within individuals with FXS is important to understand the scope of the disorder’s effects on the individual and within the family, as well as to develop treatment targets that involve and influence the dimensions of health that comprise HRQoL, including physical, social, and adaptive functioning. In clinical trials for children with FXS, assessing the HRQoL of subjects could help identify and evaluate the efficacy of the interventions beyond treating a specific symptom(s) that may present in differing intensity across youth. Likewise, HRQoL measures like the PedsQL may provide a more complete view of the health of children and their family and could be important components of natural history study to assess HRQoL over time. Based on the results from the present study, the PedsQL is a promising measure of child QoL in FXS. The measure was highly related to psychological and behavioral facets of the disorder typically studied in intervention trials for FXS.

## Figures and Tables

**Table 1:** Demographic information.

Demographics	Total		Male		Female	
Variable	Mean	*SD*	Mean	*SD*	Mean	*SD*
**Age**	15.37	11.11	15.83	11.53	14.09	9.88
**FSIQ (*n* = 113)**	49.96	24.84	42.89	22	67.1	23.16
**Verbal IQ**	50.32	26.56	42.96	23.76	68.17	24.71
**Nonverbal IQ**	49.61	24.27	42.84	21.65	66.03	22.62

**Table 2:** Mean, standard deviation, and range of PedsQL scores for present and previous studies, and significant differences between samples.

Scale	Current Sample			Online Survey Sample		Sample Differences	
	Mean	*SD*	Range	Mean	*SD*	*t* Score	*p* Value
**Total Score**	67.37	17.55	13.04–100	Not reported	-	-	-
**Physical**	71.04	23.50	9.3–100	63.48	*21.50*	1.30	0.19
**Psychosocial Summary**	65.39	17.01	11.67–100	Not reported	*-*	-	-
**Social**	59.64	26.17	0–100	47.19	21.10	2.53	0.01 *
**School**	65.43	18.53	15–100	53.96	18.80	2.15	0.03 *
**Emotional**	71.18	18.68	20–100	57.55	19.30	2.35	0.02 *

* *p* < 0.05.

**Table 3:** Correlations for PedsQL with adaptive behavior and social responsiveness.

Dimension	Total	Psychosocial	Physical	Emotional	Social	School
**Vineland Standard Scores**						
**Socialization**	0.357 **	0.251 **	0.433 ***	0.066	0.284 ***	0.261 **
**Communication**	0.242 **	0.116	0.364 ***	0.025	0.135	0.104
**Daily Living Skills**	0.311 ***	0.152	0.468 ***	–0.025	0.213 **	0.165
**Social Responsiveness Scale**	–0.656 ***	–0.631 ***	–0.547	–0.371	–0.371	–0.519

** *p* < 0.01; *** *p* < 0.001.

**Table 4:** Correlations for PedsQL with aberrant behaviors.

Dimension	Total	Psychosocial	Physical	Emotional	Social	School
**Irritability**	–0.48 ***	–0.42 ***	–0.446 ***	–0.318 ***	–0.34	–0.384 ***
**Lethargy**	–0.56 ***	–0.51 ***	–0.51	–0.32	–0.42	–0.52
**Stereotypy**	–0.49 ***	–0.49 ***	–0.4	–0.32	–0.44	–0.53
**Hyperactivity**	–0.33 ***	–0.36 ***	–0.22 **	–0.20 **	–0.26	–0.46
**Inappropriate Speech**	–0.27 ***	–0.22 **	–0.27 **	–0.19 **	–0.14 **	–0.26
**Avoidance**	–0.27 ***	–0.41 ***	–0.49	–0.31	–0.37	–0.31
**Total**	–0.56 ***	–0.53 ***	–0.47 ***	–0.33	–0.33	–0.56

** *p* < 0.01; *** *p* < 0.001. Reliabilities are Cronbach’s alpha coefficients.
